# Supplementary figures and images for: Mesquite bugs, other insects, and a bat in the diet of pallid bats in southeastern Arizona
Source: PeerJ. 2018 Dec 4;6:e6065. doi: 10.7717/peerj.6065 (PMC6284427; doi:10.7717/peerj.6065)

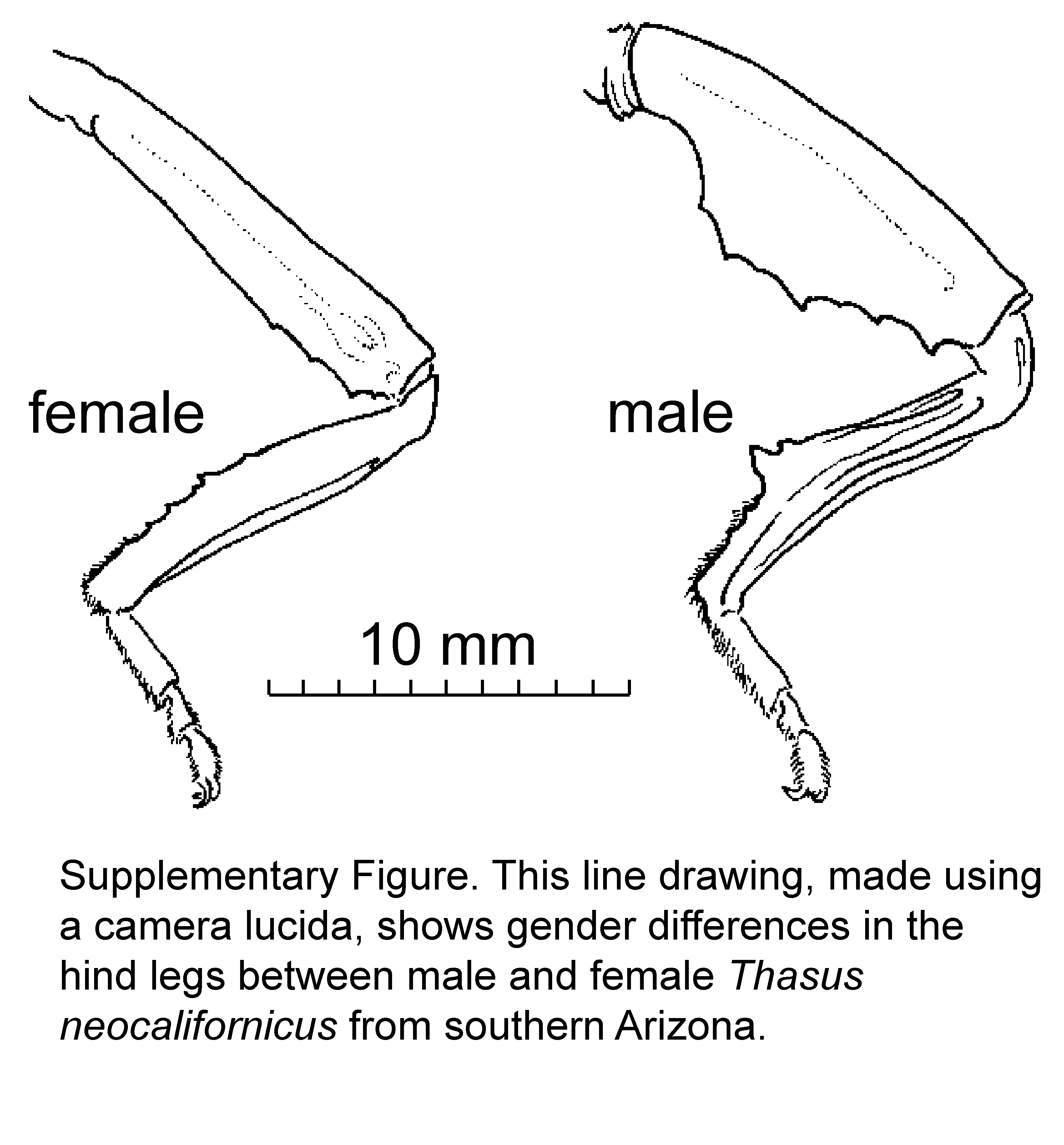

Supplement: Figure S1 [file peerj-06-6065-s001.png]
