# Supplementary material for: Mesquite bugs, other insects, and a bat in the diet of pallid bats in southeastern Arizona
Source: PeerJ. 2018 Dec 4;6:e6065. doi: 10.7717/peerj.6065 (PMC6284427; doi:10.7717/peerj.6065)
Supplement: Table S1 [file peerj-06-6065-s002.docx]

Supplementary Table 1. This table provides dimensions of guano pellets of several bat species.

*Antrozous pallidus* reddish guano pellet dimensions (to nearest 0.5 mm), soil-piping cavities, Cienega Natural Preserve, from field notes 6 January 2001

diameter length

3 8

3 10

3 9

3 5

3 8

2 5

3.5 8

3 10.5

3.5 7

3.5 11

3.5 9

2.5 5

3 12

3.5 8

3 10

3 8

3.5 6.5

3 5

3 8

3.5 8

3 6

3 7

2.5 5

=70.5/23 = 179/23

n= 23

mean diam.= 3.065

mean length= 7.783

*Myotis velifer* (Oklahoma)

2.5 6.5

2 3

1.5 6

2 5

2 3

2 3

2.5 3

2 5

2.5 4

2 3.5

1.5 3

2 3

2 5

2 3.5

= 28.5/14 = 56.5/14

n= 14

mean diameter = 2.036

mean length = 4.036

*Corynorhinus townsendii* (Oklahoma)

2 5.5

2 3

2 3

2 4

2 3

1.5 4

2 4

= 13.5/7 = 26.5/7

n= 7

mean diam. = 1.929

mean length = 3.786
