# Supplementary material for: Mesquite bugs, other insects, and a bat in the diet of pallid bats in southeastern Arizona
Source: PeerJ. 2018 Dec 4;6:e6065. doi: 10.7717/peerj.6065 (PMC6284427; doi:10.7717/peerj.6065)
Supplement: Supplemental Information 1 [file peerj-06-6065-s003.docx]

Supplemental File:

This file lists defensive chemicals of *Thasus* (mesquite bug) and online sources from which the information was taken on the dates indicated.

Wiley Online Library. 2012. 1-Hexanol [MAK Value Documentation. 1998]. The MAK Collection for Occupational Health and Safety. 284-290.

<http://onlinelibrary.wiley.com/doi/10.1002/3527600418.mb11127kske0009/full>

(accessed 8 April 2014) doi: 10.1002/3527600418.mb11127kske0009

**1-hexanol** is not lethal to laboratory rats

readily metabolizes to 2-ethyl-1-hexanol, which is a moderate skin, eye, and mucous membrane irritant in laboratory animals

Bibra toxicology advice and consulting. Toxicity profile for 2-ethyl-1-hexanol (1990)

<http://www.bibra-information.co.uk/profile-129.html>

(accessed 8 April 2014)

**n-hexyl acetate** has relatively low toxicity

**1-hexanal** The vapor is irritating to eyes, nose, throat , and skin of humans

ACToR [Aggregated Computational Toxicology Resource] Database: actor_2012q1 Chemical Summary: hexanal (66-25-1)

<http://actor.epa.gov/actor/GenericChemicalPdfServlet?casrn=66-25-1>

(accessed 8 April 2014)
