# Supplementary material for: Mesquite bugs, other insects, and a bat in the diet of pallid bats in southeastern Arizona
Source: PeerJ. 2018 Dec 4;6:e6065. doi: 10.7717/peerj.6065 (PMC6284427; doi:10.7717/peerj.6065)
Supplement: Supplemental Information 2 [file peerj-06-6065-s004.docx]

Supplemental File:

This file contains identifications of insects processed and eaten by Antrozous pallidus in soil-piping cavities.

Localities and Cavities:

Caretaker’s house porch awning 3590’ elev. (photos of A. pallidus night-roosting up to 50+; summer usage precedes 1994)

Gully system A: Pallid Bat Tunnell (PB Tunnel) 3470’ (episodic roof collapse occasionally covers parts)

Gully system B: “Y” cave 3470 ’ (bats use first noticed 1995)

Horseshoe, gully system III: Tunnel-T cave 3385’ (continuous red guano record since 1994, w/ sightings of Antrozous up to 10)

Sample Taxon Body part(s) (no. of pcs.) Tooth marks apparent?

Hemiptera

1A Thasus neocalifornicus heads w/ antennae (5)

mesquite bug

(Coreidae)

1B “ additional antennae (9)

1C “ thorax (dorsal portion) (8) Yes

thorax w/ front & hind wings (1)

abdomens (3)

1D “ forewings (272)

1E “ hindwings (40)

1F “ legs: fore & mid legs (9)

hind femurs (40-inflated 27, not inflated 13)

hind tibiae (52-ridged, bent w spur 40; straight, no spur 12)

Coleoptera

2. Chrysina/Plusiotis gloriosa elytron (1) Yes

jewel scarab

(Scarabaeidae)

3 (#27) Stenomorpha head & thorax (1) Yes on elytron

darkling beetle

(Tenebrionidae) same as no. 12

4 ?Tenebrionidae elytra (3) Yes (one on distal half; one on proximal edge)

5 ?Scarabaeidae elytra (2) Yes (on proximal quadrant)

?Cotalpa lanigera

6 Scarabaeidae A elytron (1) Yes

7 Polyphylla decimlineata elytra (4) Yes, barely

ten-lined fruit chafer

(Scarabaeidae)

8A ?Tenebrioninae elytra (crushed)(1) No

?Eleodes

8B ?Carabidae leg (unassociated w/ 8A) (1)

9 ColeopteraB elytron (1) Yes (prox half chewed up)

10 ScarabaeidaeB elytron (1) Yes (anterior, near midline)

11 cf. Calosoma scrutator elytron fragment (1) No

fiery searcher

12 Stenomorpha elytron (1) Yes (very chewed up)

(same as no. 3)

13 Coleoptera hindwing (1) No

Orthoptera

14 Tettigoniidae forewings (5) No

15 “ leg (1) No

16 hindwings (5) No

17 forewing (1) No

18 hindwings? (2) Yes?

19 forewing fragments (2) No

20 hindwing (1) No

21 ?Orthoptera wing (1) No

22 ?order part? (1)

23 “ sclerites transluscent No

Lepidoptera

24 forewings (2) No

25 Eumorpha forewings (5) No

(Sphingidae)

26 Hyles lineata forewings (3) No

white-lined sphinx

(Sphingidae)

27 “ hindwings (3) No

28 check ?Sphingicampa hubbardi forewing (1) No

mesquite moth

29 Eumorpha hindwing (1) No

(Sphingidae)

30 “ hindwing (1) No

31 ?Catocala hindwing (1) No

underwing moth

(Noctuidae)

32 hindwings (2) No

33 forewing (1) No

34A check ?Sphingicampa forewing (1) No

34B wings (2) No

Sample Taxon Body parts No. of pcs.

Hemiptera

Thasus neocalifornicus forewings 127

hindwings 56

head & antennae 8

thorax 18

hind femurs 31 (11 inflated; 20 uninflated)

hind tibiae 57

abdomens 5

other legs 21

Neuroptera

Myrmeleontidae forewing frag. 1

antlion hindwing 1

Odonata

Aeschnidae forewing 1

Check ?Boyeria hindwing 1

Orthoptera

Acrididae wing fragments 1

Lepidoptera

Eumorpha forewing 1

Family indet.? Wing frags. 1

Blattodea

Blattidae forewing 1

Coleoptera

?Carabidae head 1

thorax 1

Scarabaeidae head 1 No toothmarks

Thorax w horn 1 “

Dynastinae elytra 2 Yes

rhinoceros beetles legs 3 No

check Xyloryctes abdominal segments 5 “

Scarabaeidae elytron 1

“ elytron 1 No

“? Fused elytra 1 Yes

?Carabidae elytron 2 Yes

Sample Taxon Body parts No. of pcs Toothmarks?

Diptera

1 ?Tipulidae wing 1 No

crane fly

Coleoptera

2 Calosoma scrutator elytron 1 No

Abdomen & leg 1

3 Hydrophilus elytron 1

water scavenger beetle

4 ?Cotalpa elytra 2 Yes

5 Polyphylla decemlineata elytron 1 No

6 ?Tenebrionidae elytron 1 No

7 abdomen 1 No

8 elytra 2 Yes

21 hindwing 1 No

Lepidoptera

9 Hyles lineata forewing 1

10 “ forewing 1

11 hindwing 1

12 Sphingidae forewings 2

Orthoptera

13 Tettigoniidae forewings 7 Yes

14 “ hindwings 5

15 “? Forewings 1

hindwings 5

16 Schistocerca leg frag. 1

green valley grasshopper

17 ?Tettigoniidae hindlegs 4

18 forewings 19

19 hindwings 57

Hemiptera

Thasus neocalifornicus forewings 213

Head 1

Most of body 1

Hindwings 45

Hind femurs 21 (14 inflated, 7 uninflated)

Hind tibiae 22 (w spur 14, straight 8)

Abdomens 8
